# Agrin Influences Botulinum Neurotoxin A-Induced Nerve Sprouting via miR-144-agrin-MuSK Signaling

**DOI:** 10.3389/fcell.2020.00015

**Published:** 2020-01-30

**Authors:** Lin Ma, Lizhen Pan, Wuchao Liu, Ying Liu, Xuerui Xiang, Yougui Pan, Xiaolong Zhang, Lingjing Jin

**Affiliations:** ^1^Department of Interventional Radiology, Shanghai Tongji Hospital, Tongji University School of Medicine, Shanghai, China; ^2^Neurotoxin Research Center, Tongji University School of Medicine, Key Laboratory of Spine and Spinal Cord Injury Repair and Regeneration, Ministry of Education of the People’s Republic of China, Shanghai, China; ^3^Department of Neurology, Shanghai Tongji Hospital, Tongji University School of Medicine, Shanghai, China

**Keywords:** botulinum neurotoxin, nerve sprouting, agrin, muscle-specific receptor tyrosine kinase, microRNA

## Abstract

Botulinum neurotoxin (BoNT) has become a powerful therapeutic tool, and is extensively used in aesthetic medicine and in the treatment of neurological disorders. However, its duration of effect is limited, mainly owing to nerve sprouting. Inhibition of nerve sprouting to prolong the effective duration of BoNT is therefore of great clinical interest. However, appropriate interventional strategies to accomplish this are currently unavailable. In this study, we determined the role of the neurogenic regulator agrin in BoNT type A (BoNT/A)-induced nerve sprouting in a rat model. We then determined whether agrin could be used as an interventional target for prolonging the duration of effect of BoNT/A, and made a preliminary study of the upstream and downstream regulatory mechanisms by which agrin could influence the effective duration of BoNT/A. Our results showed that agrin was involved in the regulation of BoNT/A-induced nerve sprouting, and blocking of agrin function with anti-agrin antibody temporarily could delay muscle strength recovery and prolong the duration of BoNT/A effect. Moreover, agrin influenced the duration of BoNT/A effect by regulating downstream myogenic muscle-specific receptor tyrosine kinase (MuSK), and was simultaneously regulated by upstream miR-144. In conclusion, agrin could regulate BoNT/A-induced nerve sprouting through miR-144-agrin-MuSK signaling; it influences the effective duration of BoNT/A, and could find clinical application as an interventional target for prolonging the effect of BoNT/A.

## Introduction

The highly toxic botulinum neurotoxin (BoNT) is produced by neurotoxic anaerobic and spore-forming strains of bacteria of the genus *Clostridium*. BoNT is extensively used in aesthetic medicine and treatment of conditions such as cervical dystonia, spasticity, cerebral palsy, chronic migraine, and Raynaud’s syndrome (Krishnan, 2005; Chinnapongse et al., 2010; Foley et al., 2013; Grazzi and Usai, 2014; Zhang et al., 2015). Seven immunologically distinct BoNT serotypes (type A-G) have been reported, of which type A (BoNT/A) is the most widely used in clinical therapeutics.

BoNT/A selectively blocks the release of chemical transmitters at neuromuscular junctions (NMJs). However the blocking effect of BoNT/A gradually fades 3–4 months after application (Dolly and Aoki, 2006). Repeated injections are therefore usually required to maintain the therapeutic effect of BoNT/A. However, repetitive injection could cause immune responses and toxin resistance (Pellizzari et al., 1999). Thus, novel methods to prolong the duration of BoNT/A effect are of great clinical interest.

Nerve terminal sprouting triggered by BoNT/A is the main factor that limits the effective duration time of BoNT/A. Nerve sprouts continue chemical transmission and restore muscle contraction, and also restore original motor endplates by accelerating synaptic vesicle recycling (Juzans et al., 1996; Santafe et al., 2000). Therefore, inhibition of nerve sprouting could vastly improve the successful application of BoNT/A; however, it is also challenging—there are few effective interventional targets for prolonging the effect time of BoNT/A.

Agrin, a neuronal aggregating factor, is a 400–600 KD heparin sulfate glycoprotein molecule and a vital regulator of synaptic differentiation and maturation (Bezakova et al., 2001; Sanes and Lichtman, 2001; Ngo et al., 2007). Agrin is mainly synthesized by motor neurons and transported to nerve endings for release. Motor-neuron-derived agrin can activate MuSK by binding to the lipoprotein receptor related protein 4 (Lrp4), and induce the aggregation of acetylcholine receptors (AChRs) (Reist et al., 1992; Kim et al., 2008; Zong and Jin, 2013; Tezuka et al., 2014). However, there has been no research to determine whether agrin is involved in regulating BoNT/A-induced nerve sprouting. In the current study, we aimed to determine the effect of agrin on nerve sprouting induced by BoNT/A, and the interventional potential of agrin for prolonging the effect time of BoNT/A in decreasing muscle strength. Furthermore, we explored the mechanisms underlying the effects of agrin on the duration of BoNT/A effect.

## Materials and Methods

### Animals and Injections

The animals used in this study were maintained in accordance with the Guide for the Care and Use of Laboratory Animals published by the US National Institution of Health (NIH Publication No 85-23, revised 1996) and the Policy of Animal Care and Use Committee of Tongji University. Male Sprague-Dawley rats (200–220 g) and rats at the 1st, 4th, and 8th week after birth were obtained from B&K Universal Group Limited (Shanghai, China). Adult rats were fed with chow and water *ad libitum* at the Animal Center of Tongji Hospital, and maintained under controlled temperature (20–22°C) and a 12 h light/dark cycle.

Adult rats were randomly divided into three groups: control group (*n* = 21), BoNT/A group (*n* = 21), and agrin-Ab groups. BoNT/A (BOTOX^®^, Allergan, Co. Mayo, Ireland) was reconstituted in saline (NS) to a final concentration of 2 U/100 μL. Animals of the BoNT/A and agrin-Ab groups were injected unilaterally with 100 μL BoNT/A in the right gastrocnemius muscle under anesthesia with an intraperitoneal injection of pentobarbital (30 mg/kg). On the 3rd day after BoNT/A injection, the each subgroups of agrin-Ab were injected with 100 μL agrin-Ab (R&D Systems, Minnesota, CA, United States) at a dosage of 0.6, 2, 6, 20, or 60 μg respectively at once. Controls received an equivalent volume of NS injections in the right gastrocnemius muscle.

### Muscle Strength Determination

A survey system (CN102599921A) composed of a fixing device, sensing means, and data handling equipment was used to evaluate the muscle strength of the right hind limb of rat (Feng et al., 2017). Rats were lightly anesthetized with an intraperitoneal injection of pentobarbital (30 mg/kg) and secured on a special adjustable operating table invented by us (CN202036227U) on days 0 and 3, and after 1, 2, 4, 8, 10, and 12 weeks after BoNT/A injection. Stimulation (28 V over 0.4 ms) of the sciatic nerve led to contraction of the gastrocnemius and plantar flexion and rotation of a footboard, which was converted to electrical signals by a muscular tension energy transducer and recorded by the computer.

### Western Blot Assay

Tissue from the right gastrocnemius muscles of rats of each group at each time point after injection, and the spinal cords of rats 1, 4, and 8 weeks after birth were collected and homogenized in cold radioimmunoprecipitation assay lysis buffer (Beyotime, China) with 1:100 volume PMSF. After centrifugation at 1000 × *g* for 5 min at 4°C, proteins were extracted and the concentrations were assayed in duplicate by using the BCA protein assay kit (Pierce, United States). Protein samples (20 μg/lane) were separated by SDS-PAGE and transferred onto Hybond-P polyvinylidene difluoride (PVDF) membranes (Millipore, United States). After blocking in 5% (w/v) BSA (Sigma, United States) and washing with tris-buffered saline with Tween-20 (TBST), the membranes were then incubated with antibodies against agrin (R&D Systems, Minnesota, CA, United States), anti-MuSK (Abcam, United States), and anti-GAPDH (Abcam, United States) at 4°C overnight. After incubating with IRDye800-conjugated secondary antibody (Rockland, Philadelphia, PA, United States) for 1 h at room temperature and washing with TBST, images were acquired and band density was analyzed using Odyssey Infrared Imaging System (LI-COR Biosciences, United States). GAPDH was used as a loading control.

### RNA Isolation and Real-Time qRCR

Total RNA was extracted from the right gastrocnemius muscles of each group of rats at each time point after injection and spinal cords of rats 1, 4, and 8 weeks after birth by using Trizol reagent (Invitrogen, Carlsbad, CA, United States). The reaction mixture containing 1 μg RNA was reverse transcribed into cDNA by using the PrimeScript^TM^RT reagent Kit (TaKaRa, Dalian, China). For miRNA expression analysis, the total RNA (1 μg) was polyadenylated with ATP and poly A polymerase (PAP) at 37°C for 1 h in a 20-μl reaction mixture following the manufacturer’s protocol (Poly A Tailing Kit, Ambion, United States). After phenol-chloroform extraction and ethanol precipitation, the RNAs were reverse-transcribed using specific RT primers and PrimerScript Reverse Transcriptase (TaKaRa, Dalian, China). Quantitative real-time PCR was performed using SYBR Premix Ex Taq^TM^ (TaKaRa, Dalian, China), with primers specific for AGRIN, MuSK, and miRNA-144 according to the manufacturer’s protocol. Gene expression levels were calculated based on the comparative quantitative method (ΔΔC_T_ method) with GAPDH and 5S ribosome RNA as internal reference RNA. The primer sequences used were:

*AGRIN*: forward 5′-GGGAATGTTATGTGGCTTTGGTG-3′ reverse 5′-CATGAGGCAGTCTGTCCGTCAG-3′;

*MuSK*: forward 5′-GACACCCGCTACAGCATCCG-3′ reverse 5′-CACCGCTCCTCCCACTCCAT-3′;

*GAPDH*: forward 5′-GACAACTTTGGCATCGTGGA-3′ reverse 5′-ATGCAGGGATGATGTTCTGG-3′;

Rno-miR-144: forward 5′-TACAGTATAGATGATGTACTA-3′ reverse 5′ -GCTGTCAACGATACGCTACGTAACG-3′;

Rno-miR-27a: forward 5′-TTCACAGTGGCTAAGTTCCG C-3′ reverse 5′-GCTGTCAACGATACGCTACGTAACG-3′;

Rno-miR-29a: forward 5′-TAGCACCATCTGAAATCGGT TA-3′ reverse 5′-GCTGTCAACGATACGCTACGTAACG-3′;

5S ribosome RNA: forward 5′-GTCTACGGCCATACCCTGA AC-3′ reverse 5′-GCTGTCAACGATACGCTACGTAACG-3′.

### Luciferase Assay

The 3′-UTR and mutant fragment of agrin and were subcloned into the Xbal restriction site downstream of the firefly luciferase gene of the pGL3-Basic Vector (Promega, United States) as pGL3-3′-UTR. The pRL-TK vector (Promega, United States) containing *Renilla* luciferase gene was used as an internal control reporter vector. The DNA sequences of pre-miR-144, pre-miR-27a, and pre-miR-29a were amplified by PCR and subcloned into pSuper-EGFP1 vector as pSuper-144, pSuper-27a, and pSuper-29a. At 24 h post-transfection of pSuper-miRNAs vector, the constructed luciferase reporter plasmids and the pRL-TK vectors were co-transfected into HEK293T cells at a ratio of 50:1. Luciferase activity was measured 24 h after transfection by using the dual luciferase reporter assay system (Promega, United States) according to the manufacturer’s protocol. Firefly luciferase activity was normalized to *Renilla* luciferase activity.

### α-Bungarotoxin Staining

Gastrocnemius muscle samples were harvested and fixed with 4% paraformaldehyde. Cross-sections from the fixed samples were rehydrated in PBS for 5 min at room temperature. The sections of 10 μm were blocked in immunofluorescence blocking buffer (Beyotime, China) for 30 min at room temperature and incubated with staining solution containing 1 μg/mL of α-bungarotoxin conjugated with tetramethylrhodamine (Biotium Inc., Fremont, CA, United States) in immunofluorescence blocking buffer at 4°C overnight. After rinsing three times, nuclei were counterstained with 4′,6-diamidino-2-phenylindole (DAPI). Nikon instruments were used to capture fluorescence pictures.

### Statistical Analysis

All data were expressed as the mean ± standard deviation (SD). A one-way analysis of variance (ANOVA) was conducted to evaluate the data. Statistical significance was determined by Student’s *t*-test. *P* < 0.05 was considered statistically significant.

## Results

### Agrin Participates in Regulating BoNT/A-Induced Nerve Sprouting

**FIGURE 1:**
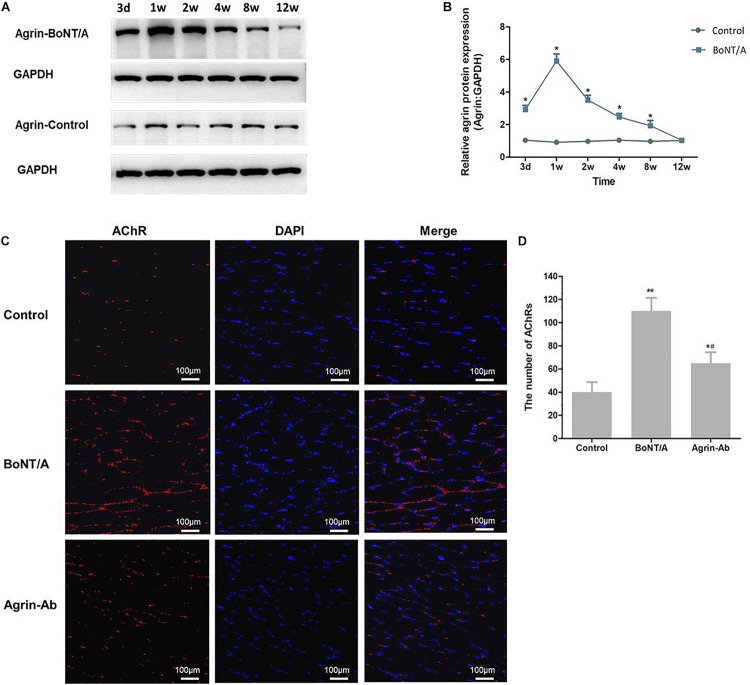
Agrin participates in regulating nerve sprouting after BoNT/A application. **(A)** Western blot analysis of agrin at NMJs after BoNT/A injection. GAPDH was used as internal control. **(B)** Quantitation of agrin protein was performed using GeneTools from SynGene software and normalized to GAPDH. *P < 0.05 versus control. **(C)** α-Bungarotoxin staining analysis of the number of AChRs after BoNT/A and agrin-Ab injection at week 1. Nuclei were counterstained with DAPI (4′-6-diamidino-2-phenylindole; blue). Images were merged as indicated. Scale bar, 100 μm. **(D)** Number of AChRs; bars represent mean ± SD of three different experiments. *P < 0.05, **P < 0.01 compared to the control group. #*P* < 0.05 compared to the BoNT/A group.

To determine whether agrin is involved in regulating nerve sprouting induced by BoNT/A, we first studied agrin expression at NMJs 3 days and 1, 2, 4, 8, and 12 weeks after BoNT/A application. Results showed that compared with the control group, the expression of agrin in the BoNT/A group began to increase on day 3, increased to the highest level at 1 week, and gradually returned to normal (control) level, suggesting that agrin might participate in the regulation of nerve sprouting after BoNT/A application (Figures 1A,B).

To confirm that agrin participates in regulating nerve sprouting, anti-agrin antibody (agrin-Ab; 20 μg) was injected into the rats’ gastrocnemius muscle after BoNT/A injection to antagonize agrin. α-bungarotoxin staining was performed to observe nerve sprouting and NMJs. We found that the number of AChRs increased significantly after BoNT/A injection in the 1st week (*P* = 0.007), and was significantly lower in the agrin-Ab group than in the BoNT/A group (*P* = 0.029; Figures 1C,D). These results indicated that agrin participated in regulating BoNT/A-induced nerve sprouting.

### Agrin Could Serve as an Interventional Target for Prolonging the Effective Duration of BoNT/A

**FIGURE 2:**
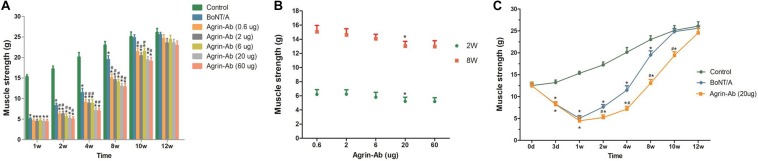
Agrin-Ab prevents the recovery of muscle strength and prolongs the duration of BoNT/A effect in a dose-dependent manner. **(A)** Muscle strength was determined by a survey system for rat lower limb nerve and muscle function at different times after injection of BoNT/A and agrin-Ab at different doses. *P < 0.05 versus the control group; #*P* < 0.05 versus the BoNT/A group. **(B)** Dose-dependent effect of agrin-Ab in maintaining the decreased muscle strength caused by BoNT/A at week 2 and week 8. Muscle strength was determined by a survey system for rat lower limb nerve and muscle function. *P < 0.05 versus the 6 μg group. **(C)** The paralytic effect of BoNT/A lasted longer after injection of 20 μg agrin-Ab. *P < 0.05, compared to the control group. #*P* < 0.05, compared to the BoNT/A group.

After concluding that agrin is involved in the regulation of BoNT/A-induced nerve sprouting, we confirmed that agrin influences the duration of BoNT/A effect by regulating nerve sprouting, and hypothesized that agrin could be used as an interventional target for prolonging the duration effect of BoNT/A. To verify our hypothesis, the function of agrin was inhibited using agrin-Ab. Various doses of recombinant agrin-Ab (0.6, 2, 6, 20, or 60 μg) were intramuscularly injected into the gastrocnemius muscle on day 3 after BoNT/A injection. As indicated in Figure 2A, muscle strength in the control group gradually increased with an increase in weight. Compared with the control group, muscle strength in the BoNT/A group were significantly lower at 3 days and weeks 1, 2, 4, and 8 after BoNT/A injection. Muscle strength in the BoNT/A group returned to the control level at week 10. However, compared with the BoNT/A group, muscle strength in the agrin-Ab groups decreased further at weeks 2, 4, 8, and 10. Moreover, muscle strength in the agrin-Ab groups reverted to the level of the control group at week 12, which was two weeks longer than that in the BoNT/A group (*P* = 0.002), suggesting that agrin-Ab can prolong the duration of effect of BoNT/A in decreasing muscle strength. Agrin-Ab showed a dose-dependent effect in maintaining the decreased muscle strength caused by BoNT/A, and there was no difference in effect between the 20 and 60 μg dosage subgroups (Figure 2B). 20 μg is therefore likely the threshold dose, and was selected for subsequent experiments (Figure 2C). These findings revealed that agrin could be used as an interventional target for prolonging the duration of effect of BoNT/A in decreasing muscle strength.

### Agrin Influences the Duration of BoNT/A Effect by Regulating Downstream MuSK

**FIGURE 3:**
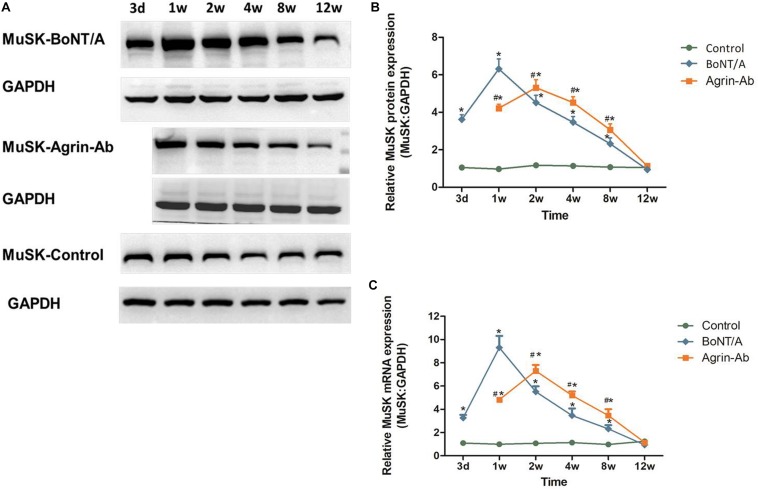
Agrin-Ab delays the increase of MuSK expression. **(A)** Protein levels of MuSK were assayed by western blot after BoNT/A and agrin-Ab injection. GAPDH was used as an internal control. **(B)** Protein quantification was normalized to GAPDH and data are presented as mean ± SD of three different experiments. *P < 0.05 compared to the control group. #*P* < 0.05 compared to the BoNT/A group. **(C)** mRNA levels of MuSK were assayed by qPCR after BoNT/A and agrin-Ab injection. GAPDH was used as an internal control. Data represent the mean ± SD of three different experiments. *P < 0.05 compared to the control group. #*P* < 0.05 compared to the BoNT/A group.

The trends observed in agrin expression after BoNT/A injection were similar to those of MuSK, which we previously reported (Jin et al., 2013), suggesting that agrin might regulate MuSK expression. Therefore, we speculated that agrin influences nerve sprouting via agrin-MuSK signaling and thereby influences the effective duration time of BoNT/A. After agrin-Ab (Ab 20 μg) injection, MuSK mRNA and protein levels at different times after BoNT/A injection were analyzed by qPCR and western blot. MuSK expression increased in the 1st week, and reached its peak in the 2nd week in the agrin-Ab group, which was later than that in the BoNT/A group (Figures 3A–C). These findings, combined with our previous findings (Jin et al., 2013; Guo et al., 2015), indicated that agrin influences the effective duration time of BoNT/A by regulating downstream MuSK.

### Agrin Expression Is Regulated by Upstream miR-144

**FIGURE 4:**
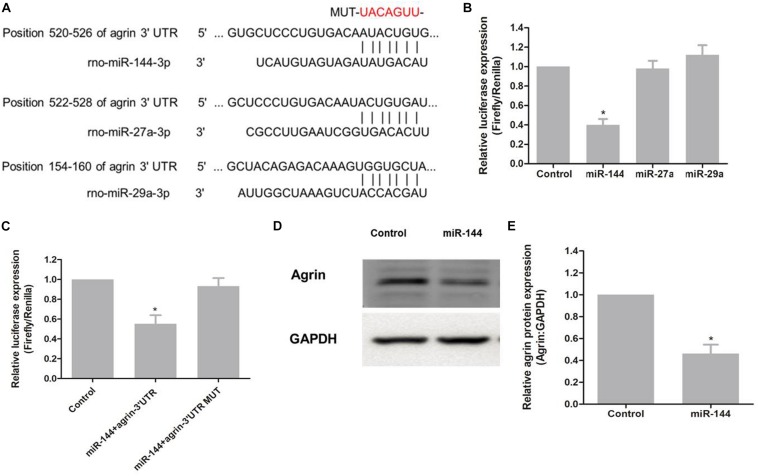
miR-144 regulates agrin expression by directly targeting the 3′ UTR of agrin mRNA. **(A)** Schematic representation of three putative miRNA-binding sites on the agrin 3′ UTR sequence. **(B)** The 3′ UTR fragment of agrin was cloned into the Xbal restriction site downstream of the firefly luciferase gene of the pGL3-Basic vector. Luciferase activity of each sample was measured 48h after transfection and normalized to *Renilla* luciferase activity. pGL3-Basic vector was used as control. Graph error bars indicate SD calculated from at least three independent experiments. *P < 0.05, compared with the control. **(C)** Co-transfection of pSuper-144 with the firefly luciferase reporter gene linked to the wild-type and mutant-type sequence of miR-144 binding site within the agrin 3′ UTR. Luciferase activity was measured and normalized to phRL-TK activity. Three independent experiments were performed and data are presented as mean ± SD. *P < 0.05, compared with the control. **(D)** Protein level of agrin was assayed by western blotting. GAPDH was used as an internal control. **(E)** Protein expression was normalized to GAPDH. *P < 0.05, compared to the control group.

To elucidate the regulatory microRNAs (miRNAs) of agrin, we searched for putative miRNAs by using prediction algorithms (miRDB, miRanda and TargetScan). Three miRNAs with high scores, miR-144, miR-27a, and miR-29a, were selected as candidates (Figure 4A). Luciferase assay results showed that luciferase activity was reduced by 40% in HEK 293T cells transfected with pSuper-144 compared with the control pSuper-EGFP1, pSuper-27a, and pSuper-29a, suggesting that miR-144 could suppress gene expression by binding sequences at the 3′ UTR of agrin (*P* = 0.006; Figure 4B). To determine whether agrin is a direct target of miR-144, we mutated the seed sequence of miR-144 in the agrin 3′ UTR shown in Figure 4A. Co-transfection of pSuper-144 with the firefly luciferase reporter gene linked to the wild-type segment of the agrin 3′ UTR strongly repressed luciferase activity, and the luciferase activity in the group with the mutant-type (MUT) segment of the agrin 3′ UTR was rescued (Figure 4C). These results showed that miR-144 could directly target the 3′ UTR of agrin mRNA.

To determine whether miR-144 regulates agrin expression, pSuper-144 was transfected into primary neural progenitor cells according to a previously reported protocol (Zhang et al., 2013), and the expression of agrin was examined. Agrin protein levels notably decreased after pSuper-144 over-expression compared with the control group (*P* = 0.006*;* Figures 4D,E). These results suggested that miR-144 regulates agrin expression by targeting the 3′ UTR of agrin mRNA.

### The Regulatory Effect of Agrin on Nerve Sprouting Is Regulated by Upstream miR-144

**FIGURE 5:**
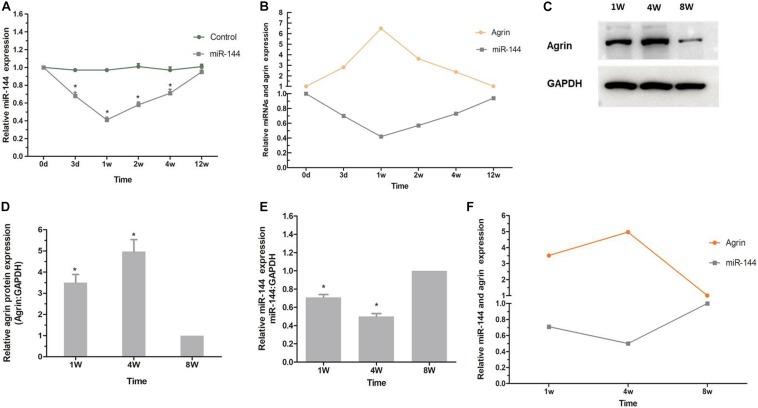
miR-144-agrin signaling participates in regulating NMJ formation. **(A)** Real time RT-PCR analysis of miR-144 expression after BoNT/A injection. *P < 0.05, compared with the control group. Data represent mean ± SD of three different experiments. **(B)** The relative trends of miR-144 and agrin expression (BoNT/A group/Control group). **(C)** The relative expression level of agrin from rats 1, 4, and 8 weeks after birth analyzed by western blot. GAPDH was used as an internal control. **(D)** Protein levels were normalized to those of GAPDH. *P < 0.05, compared to the control (8W) group. **(E)** The relative expression level of miR-144 from rats 1, 4, and 8 weeks after birth analyzed by real time RT-PCR assay. GAPDH was used as an internal control. *P < 0.05, compared to the control (8W) group. **(F)** The relative trends of miR-144 and agrin expression (BoNT/A group/Control group).

To determine whether the regulatory effect of agrin on nerve sprouting after BoNT/A injection is regulated by upstream miR-144, we examined the expression of miR-144 in the rats’ spinal cord anterior horn. Compared to miR-144 expression in the control group, miR-144 expression in the BoNT/A group began to decrease on day 3, decreased to the lowest level at 1 week, and gradually returned to normal (control) level. This was opposite to the trend observed in agrin protein expression (Figures 5A,B), indicating that the regulatory effect of agrin on nerve sprouting is regulated by upstream miR-144.

To confirm that miR-144-agrin signaling is involved in regulating the development of original motor endplates, the expression of miR-144 in spinal cord anterior horn and the expression of agrin in the gastrocnemius muscle from the rats were examined 1, 4, and 8 weeks after birth. As shown in Figures 5C,D, the expression of agrin protein gradually increased after birth, increased to the highest level at the 4th week, and then decreased to the normal level (8w), which corresponds to the development of original motor endplates (Grinnell, 1995). Further, miR-144 expression showed a contrasting trend to that of agrin expression (Figures 5E,F). These results suggested that miR-144-agrin signaling participates in regulating NMJ formation in nerve sprouting and the development of original motor endplates.

## Discussion

In this study, we revealed that agrin plays a role in regulating BoNT/A-induced nerve sprouting via miR-144-agrin-MuSK signaling, which influences the effective duration time of BoNT/A. Blocking agrin function with agrin-Ab temporarily could delay muscle strength recovery and prolong the duration of effect of BoNT/A. Agrin therefore shows potential as an interventional target for prolonging the effect time of BoNT/A in decreasing muscle strength. Our work revealed the role and the underlying mechanism of agrin in the process of nerve sprouting after BoNT/A application, and simultaneously explored a novel interventional target for prolonging the effect of BoNT/A.

Effective duration time is a vital factor that limits the clinical applications of BoNT/A. There have been several attempts to prolong the duration of BoNT/A effect, including usage of higher dosages, high-volume preparation, and electrical stimulation post-injection (Dressler, 2004; Rha et al., 2008; Boyle et al., 2009; Hu et al., 2009). However, the results of these approaches were unsatisfactory. Novel methods to prolong the effect of BoNT/A remain elusive. BoNT/A-induced nerve sprouting is the main factor that reduces the effect time of BoNT/A; inhibiting nerve sprouting could potentially prolong the effect of BoNT/A. The extent and length of nerve sprouting triggered by BoNT/A could be reduced by treatment with antibodies to insulin-like growth factor 1 (IGF-1) or neural cell adhesion molecule (NCAM) (Booth et al., 1990; Schafer and Wernig, 1998; Rind and von Bartheld, 2002; Apel et al., 2010). Our group verified that the duration of BoNT/A effect could be prolonged by inhibition of IGF-1 or NCAM function with IGF-1 or NCAM antibodies (Jin et al., 2013; Guo et al., 2015). These findings provide a basis to develop novel approaches to prolong the effect of BoNT/A.

Although agrin has been proved to have a vital role in the development of original motor endplates, there are no reports on the involvement of agrin in nerve sprouting. In this study, we found that the expression of agrin in the BoNT/A group began to increase on day 3, increased to the highest level at 1 week, and gradually returned to the normal level. The trends of agrin expression after BoNT/A application were similar to those in MuSK, which we reported previously (Jin et al., 2013; Guo et al., 2015). Moreover, the increase in nerve sprouting was significantly lower in the agrin-Ab group than in the BoNT/A group (*P* = 0.029). These results indicate the regulatory role of agrin in BoNT/A-induced nerve sprouting.

Some studies have shown that agrin-Ab can block aggregation of AChRs at original motor endplates on muscle surface (Reist et al., 1992; Gautam et al., 1996), however there have been no studies on whether agrin-Ab could prevent the recovery of NMJs and prolong the effect of BoNT/A. In this study, agrin-Ab was used to antagonize agrin after BoNT/A injection, and muscle strength was detected at different times. Compared with the BoNT/A group, muscle strength in the agrin-Ab groups declined more at weeks 2, 4, 8, and 10, and took two weeks longer to recover to normal levels. Although the effect of exogenous IgG protein did not exclude in this study, we did a dose effect curve for the agrin-Ab, and the results displayed that agrin-Ab showed a dose-dependent effect in maintaining the decreased muscle strength caused by BoNT/A (Figures 2A,B). The results still provide us the important message that agrin-Ab could prevent the recovery of muscle strength and prolong the effect of BoNT/A. Even so, exogenous IgG protein is still a factor that has to be eliminated in the future. Our results confirmed the interventional potential of agrin in prolonging the effect of BoNT/A in decreasing muscle strength, and revealed a novel potential interventional target for extending BoNT/A effects.

The trends of agrin expression after BoNT/A application were similar to those of MuSK. Therefore, we speculated that agrin participates in regulating nerve sprouting via agrin-MuSK signaling. Then we conducted further experiments that shows inhibition of agrin with agrin-Ab delays upregulation of MuSK, and thereby suppresses BoNT/A-induced nerve sprouting. Previous studies have shown that agrin binds to the first β-propeller domain of Lrp4, which evidently induces a conformational change in Lrp4 and enhances the interaction between Lrp4 and MuSK (Zhang W. et al., 2011; Zong et al., 2012; Hubbard and Gnanasambandan, 2013). Therefore, based on previous research findings, our research results could reflect the regulatory role of agrin-Lrp4-MuSK signaling in BoNT/A-induced nerve sprouting and the function recovery of NMJs after injection of BoNT/A.

Currently, there is no report on the upstream regulatory signaling of agrin. miRNAs, a vital class of upstream regulatory factors of gene expression, negatively regulate gene expression at the post-transcriptional level by binding to the 3′ UTR of target mRNAs. miRNAs are broadly involved in various biological processes including development, cell differentiation, and apoptosis *in vivo* and *in vitro* (Bartel, 2004; Ouellet et al., 2006; Wang et al., 2018). However, the regulatory miRNA of agrin has not been reported. In this study, we searched for putative miRNAs by using common prediction algorithms (miRDB, miRanda, and TargetScan) and selected three miRNAs with high scores in at least 2 algorithms simultaneously, as reported previously (Gong et al., 2015; Jiang et al., 2019). Subsequent studies indicated that only miR-144 could regulate the expression of agrin, and miR-144/agrin/MuSK axis might regulate NMJ formation in nerve sprouting and the development of original motor endplates. Zhang H.Y. et al. (2011) reported that miR-144 was down-regulated following sciatic nerve transection. Rau et al. (2010) also found that miR-144 was down-regulated after sciatic nerve denervation. We speculated that the secretion of agrin was blocked after sciatic nerve denervation, which activated the upstream signals and caused downregulation of miR-144. These findings therefore indirectly support our results. The current study showed, for the first time, that agrin could be targeted by miR-144 in neurons. Future *in vivo* and *in vitro* studies are required to elucidate the regulatory role of miR-144/agrin/MuSK signaling in the development of NMJs.

## Conclusion

Agrin is a crucial neurogenic regulator in the development of original motor endplates; however, its role in nerve sprouting remains unclear. In this study, we demonstrated that agrin participates in regulating BoNT/A-induced nerve sprouting, which limits BoNT/A efficacy, and revealed a novel mechanism of nerve sprouting. In addition, we confirmed that agrin-Ab could prevent the recovery of muscle strength and prolong the duration of effect of BoNT/A, indicating that agrin could be used as an interventional target for prolonging the duration of effect of BoNT/A in decreasing muscle strength.

## Data Availability Statement

All datasets generated for this study are included in the article/supplementary material.

## Ethics Statement

The animal study was reviewed and approved by the Committee of Tongji University.

## Author Contributions

LM and LJ conceived and designed the experiments. WL, YL, and XX performed the experiments. YP and XZ performed informatics analysis. LM, LP, and LJ wrote the manuscript. All authors read and approved the final manuscript.

## Conflict of Interest

The authors declare that the research was conducted in the absence of any commercial or financial relationships that could be construed as a potential conflict of interest.
